# A Mini Review of the Zoonotic Threat Potential of Influenza Viruses, Coronaviruses, Adenoviruses, and Enteroviruses

**DOI:** 10.3389/fpubh.2018.00104

**Published:** 2018-04-09

**Authors:** Emily S. Bailey, Jane K. Fieldhouse, Jessica Y. Choi, Gregory C. Gray

**Affiliations:** ^1^Duke Global Health Institute, Duke University, Durham, NC, United States; ^2^Division of Infectious Diseases, Duke University School of Medicine, Durham, NC, United States; ^3^Global Health Research Center, Duke-Kunshan University, Kunshan, China; ^4^Emerging Infectious Diseases Program, Duke-NUS Medical School, Singapore

**Keywords:** emerging viruses, respiratory viruses, influenza viruses, adenoviruses, coronaviruses, enteroviruses, one health

## Abstract

During the last two decades, scientists have grown increasingly aware that viruses are emerging from the human–animal interface. In particular, respiratory infections are problematic; in early 2003, World Health Organization issued a worldwide alert for a previously unrecognized illness that was subsequently found to be caused by a novel coronavirus [severe acute respiratory syndrome (SARS) virus]. In addition to SARS, other respiratory pathogens have also emerged recently, contributing to the high burden of respiratory tract infection-related morbidity and mortality. Among the recently emerged respiratory pathogens are influenza viruses, coronaviruses, enteroviruses, and adenoviruses. As the genesis of these emerging viruses is not well understood and their detection normally occurs after they have crossed over and adapted to man, ideally, strategies for such novel virus detection should include intensive surveillance at the human–animal interface, particularly if one believes the paradigm that many novel emerging zoonotic viruses first circulate in animal populations and occasionally infect man before they fully adapt to man; early detection at the human–animal interface will provide earlier warning. Here, we review recent emerging virus treats for these four groups of viruses.

## Introduction

**Figure 1:**
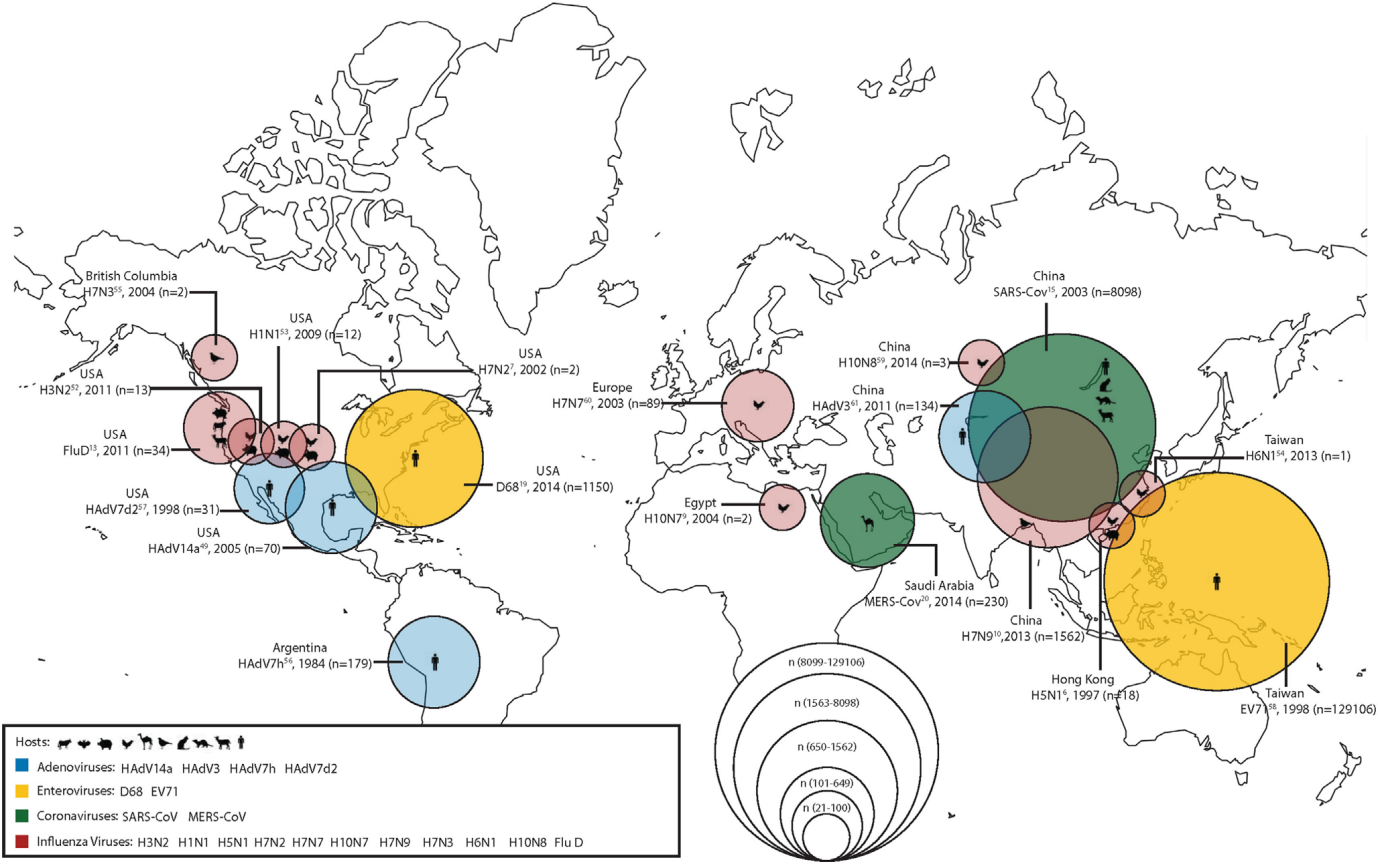
The geographical location of first detections (with known reservoirs) for recently emerged adenoviruses (Ads), enteroviruses (EVs), coronaviruses, and influenza viruses. Zoonotic (coronaviruses and influenza viruses) and non-zoonotic viruses (Ads and EVs) are shown. For zoonotic viruses, the hosts range from cattle, bats, chickens, camels, wild birds, cats, ferrets, goats, and humans (from left to right). The different sizes of the circles represent the number of human cases during the first outbreaks of the emerging respiratory viruses. Human cases of adenoviral infections are shown in blue; human cases of enteroviral infections are shown in yellow; human cases of coronaviral infections are shown in green; and human cases of influenza viral infections are shown in red.

During the last two decades, scientists have grown increasingly aware that viruses are emerging from the human–animal interface. In order to combat this increasingly complex problem, the One Health approach or initiative has been proposed as a way of working across disciplines to incorporate human, animal, and environmental health. Of particular concern are emerging respiratory virus infections; in a recent seminar given by the National Institute of Health on emerging and re-emerging pathogens, nearly 18% were respiratory viruses (1). Among the recently emerged respiratory pathogens contributing to the high burden of respiratory tract infection-related morbidity and mortality, displayed graphically in Figure 1, are influenza viruses, coronaviruses, enteroviruses (EVs), and adenoviruses (Ads). In this report, we summarize the emerging threat characteristics of these four groups of viruses.

## Zoonotic Influenza

### Introduction and Epidemiology

Influenza viruses are RNA viruses that are members of the orthomyxovirus family and classified into four types: A, B, C, and D (2). As shown in Table 1, these four types of viruses are characterized by their immunologically distinct nucleoprotein and matrix protein antigens. Influenza A and B viruses consist of hemagglutinin (HA), which binds a sialic acid receptor, allowing the virus to enter the host cell, and neuraminidase (NA), which cleaves the sialic acid to release the virus. Similarly, influenza C and D viruses contain HA-esterase fusion glycoproteins that also allow for the attachment of viral and cellular membranes. Antigenic shifts (influenza A only) in HA, NA, and the HA-esterase proteins contribute to the generation of novel viral strains. The host range of influenza viruses includes humans, birds, pigs, bats, and other livestock animals such as cattle and goats. The network of the influenza viral transmission is complex with both inter- and intraspecies transmission. As the viruses continue to change in their genetic sequences, ongoing research is imperative in investigating the ecology of these viruses at the human–animal interface to control further spread of infections and prevent the risk of future pandemics.

**Table 1:** Characteristics of influenza viruses.

Characteristics	Influenza A	Influenza B	Influenza C	Influenza D
Virus structure	Enveloped Eight gene segments Hemagglutinin (HA) and neuraminidase (NA) glycoproteins		Enveloped Seven gene segments HA–esterase fusion glycoprotein	

Epidemiology	Antigenic shift and drift	Only antigenic drift	Only antigenic drift	Only antigenic drift

Known hosts	Domestic and wild animals and avian species	Humans, pigs, and seals	Humans and pigs	Humans and livestock (cattle, pigs, and goats)

Clinical manifestation	Pandemic potential could cause high mortality	Can cause severe disease in elderly and high-risk populations	Mild seasonal disease predominantly in children	Mild disease in humans across ages

Available diagnostics	Rapid diagnostic tests (i.e., antigen detection); rapid molecular assays (nucleic acid detection); immunofluorescence; and cell culture		Real-time PCR and cell culture	

Treatments	NA inhibitors: zanamivir, oseltamivir, and peramivir. Laninamivir investigational in USA.	NA inhibitors: zanamivir, oseltamivir, and peramivir. Laninamivir investigational in USA.	Supportive symptom management	Supportive symptom management
	Adamantanes: amantadine and rimantadine (notrecommended in USA due to resistance)			

### Influenza A

#### Swine Influenza

##### H3N2

Influenza A virus H3N2 subtypes are frequently reported in swine, avian, and canine hosts that are responsible for highly infectious respiratory diseases in pigs and have been examined as a potential cause of influenza in humans. One study, examining the role of IAV in pigs at USA agricultural fairs, reported an average influenza A prevalence of 77.5% among 161 swine across all seven fairs (3). The genomic sequences of the viruses isolated from the swine were ≥99.89% similar to the H3N2 viruses isolated in humans. At these fairs, IAVs were detected at least 1 day before symptoms of the virus were observed in humans, indicating that H3N2 was transmitted from pigs to humans in this case.

##### H1N1

Since 2009, H1N1 virus has posed a significant threat to livestock workers and the greater community and has now become a seasonal influenza virus which circulates in humans. To explore the role of swine production facilities in the development of new swine-like influenza viruses, the spatiotemporal association between weekly influenza-like illnesses (ILIs) in humans and the location of pig farms was investigated in North Carolina over four influenza seasons (4). Analyses showed that the years of H1N1 pandemic, 2009–2010 and 2010–2011, were closely related with earlier peaking of ILI cases. These findings suggest that increased exposure to pigs was associated with earlier observations of the greatest number of human H1N1 cases.

In China, the transmission of influenza A between humans and pigs in six farms is being examined using a One Health approach, taking into consideration the interconnectedness of humans, animals, and the environment (5). Findings suggest that both A(H1N1)pdm09-like and swine-lineage H1N1 and swine-lineage H3N2 viruses are circulating in swine workers and that these viruses likely reassort and cross species within the pig farms; as such, additional research is needed to understand the relationship between cross species transmission of viruses in humans and pigs.

#### Avian Influenza

Avian influenza viruses are the largest group of influenza A viruses reservoired in aquatic birds or poultry. Although infrequently transmitted to humans, many cases have now been reported. Human infection with avian influenza can lead to serious health conditions, including death. The first outbreak of an IAV strain, H5N1, in humans occurred in Hong Kong SAR, China, in 1997, infecting 18 humans (6). The first identified cases of human infection with H7N2, another avian influenza, occurred in North America with two human cases reported in 2002 (7). Another variation of the virus, H7N7, was the first avian influenza strain reported in Europe; it infected 89 humans in 2003. In 2004, the first human cases of H10N7 infections were observed in Africa (8). It is important to note that H5N1 virus outbreak occurred again in 2004, 7 years after its first outbreak in humans, infecting more than 650 humans and causing more than 386 deaths worldwide (9). The avian influenza viruses have continuously evolved, causing serious infections among humans across the world.

##### H7N9

H7N9 virus, a sporadic subtype of an avian influenza A virus, was first reported in humans in China in 2013. Since the first outbreak, China has been experiencing epidemics annually, with a cumula-tive number of 1,562 reported cases, 40% of which have led to deaths as of September 2017 (10). The incidence of the H7N9 infections has been increasing in both humans and poultry and in 2017 alone 764 infections have been reported (11). Although H7N9 was first recognized as a low pathogenic avian influenza, two divergent lineages were detected in 2016—including a highly pathogenic avian influenza variant (12). According to the Center for Disease Control and Prevention (CDC), H7N9 is now recognized as the virus with the greatest potential to cause a pandemic due to its rapid genetic changes over the last 5 years. This further supports the need to improve disease control strategies and increase efforts to develop an effective vaccination strategy in the future as the spread of the H7N9 infection poses a threat to the poultry business.

#### Influenza D

Influenza D virus (IDV) is a novel influenza virus that is structurally different from the other influenza viruses. IDV was first isolated in 2001 from pigs in USA and since the first report, viral infection has been reported in various locations in USA, Europe, and Asia. In a serological study, cattle workers and non cattle-exposed adults in Florida were screened for IDV antibodies (13). Of the cattle workers, 97% of IDV seroprevalence was observed, while less than 20% was observed in non-cattle-exposed adults, suggesting a greater risk of IDV infection for cattle workers. During a swine respiratory disease outbreak in Northern Italy in 2015, the IDV genome was detected and isolated in both pigs and cattle herds (14). The viral genome isolated from the pigs was closely related to the viral genome isolated in USA in 2011. Additionally, the archived serum samples from 2009 had lower IDV antibody titers compared to the serum samples collected in 2015. These findings suggest that the incidence of IDV infections in pigs may have increased over time, and therefore, IDV may pose a public health threat to the community.

## Zoonotic Coronavirus

### Introduction and Epidemiology

Coronaviruses are single-stranded, RNA viruses with a large genome in which mutations are very common. There are six human types of coronavirus: 229E, OC43, NL63, HKU1, which are often associated with mild upper respiratory tract infections, as well as the virus causing severe acute respiratory syndrome (SARS-CoV) and Middle East respiratory syndrome (MERS-CoV), both of which are zoonotic and have previously caused human disease. Interspecies transmission and the resulting emergent coronaviruses have been important factors in emerging respiratory disease as coronaviruses are known to infect feline, swine, canine, and bat species. Both MERS-CoV and SARS-CoV emerged from animal reservoirs and are now increasingly important respiratory virus threats.

### SARS-CoV

SARS-CoV first emerged as a global problem in 2003, when China informed the World Health Organization (WHO) of 305 cases of atypical pneumonia in Guangdong Province. The WHO estimates that a total of 8,098 people worldwide became sick with SARS-CoV during the 2003 outbreak, and of them 774 died (15). In USA, only eight people had laboratory evidence of SARS-CoV infection and, in all cases, infection was travel related. The secondary spread of SARS-CoV was characterized by transmission between patients and nurses, requiring intervention from hospital infection control (16). Horseshoe bats are the host reservoir for SARS-CoV, but it is also postulated that other intermediate hosts such as civet cats, domestic cats, rodents, and swine may play an important role in transmission (17, 18). Recently, it has also been suggested that bats may play a role in the direct human transmission as bat SARS-like coronaviruses have been identified in some species (19).

In the past decade, teams from the Sabin Vaccine Institute and Baylor College of Medicine have been working toward the development of a vaccine for SARS-CoV. Although initial reports indicated that a vaccine may be ready for human clinical trials in 2017, progress has been slow and few human SARS vaccine trials have been conducted to date.

### MERS-CoV

Middle East respiratory syndrome was first recognized in Saudi Arabia in 2012. Many cases were linked to travel to or residence in countries in and near the Arabian Peninsula. Symptoms include severe acute respiratory illness with fever, cough, and shortness of breath. There is limited human-to-human transmission of MERS-CoV, but exposure to camels is a risk factor for infection, with seroprevalences 15–23 times higher in camel exposed individuals (20). Despite this, major health care-associated transmission of MERS-CoV was reported in the Middle East and Korea, with outbreaks characterized by interhospital spread related to overcrowding and a lack of personal protective equipment (21). The total number of worldwide cases reported to the WHO as of January 9, 2017 was 2,067 MERS-CoV cases (22).

The cocirculation of CoVs in its animal reservoirs (camels and bats) raises important questions about the evolution of MERS-CoV. In a study conducted between 2014 and 2015 in Saudi Arabia, researchers found that dromedary camels share three CoV species with humans, including betacoronavirus 1, MERS-COV, and a CoV 229E-related virus (23). With the aim of reducing MERS-CoV transmission to humans, Haagmans et al. developed a vaccine for camels using a poxvirus vehicle (24). This vaccine has significantly reduced virus excretion among camels and conferred cross-immunity to camelpox infections (25).

## Enteroviruses

### Introduction and Epidemiology

Enteroviruses are small, positive-sense, single-stranded RNA viruses in the Picornaviridae family. There are 12 species of EVs found globally, including EV A-J (EV-A, B, C, D, E, F, G, H, and J) and rhinovirus A–C (RV-A, B, and C). With low replication fidelity and frequent recombination, EVs have viral genetic diversity and a potential for cross-species infection. In February 2013, the International Committee on Taxonomy of Viruses (ICTV) approved changes to EV and rhinovirus species names after many of the human EV species were identified and isolated in non-human hosts. Based on an analysis of Picornaviridae hosts listed in the ICTV database and subsequent studies of EV infection in non-human primates, there is growing evidence to indicate a potential for future zoonotic transmission between animals and humans (26, 27). Among the most important emerging respiratory viruses are EV68, EV71, coxsackieviruses, echoviruses, rhinoviruses, and polioviruses.

Enterovirus transmission occurs year-round, with seasonal peaks occurring in the summer and fall (June–October). Infants less than 1 year of age are most susceptible to infection, and males are at an increased risk for infection until the age of 20 years (28). The predominant mode of transmission is through a direct or indirect fecal–oral route; however, certain serotypes are transmitted *via* the respiratory route, in tears, and *via* fomites (29). Immunity to EVs is serotype specific with most causing mild respiratory infections.

### Rhinoviruses

Rhinoviruses are small, single-stranded RNA viruses in the picornavirus family that are responsible for more than half of all upper respiratory tract infections. In addition to exacerbating asthma and chronic obstructive pulmonary disease, rhinoviruses have also been associated with acute respiratory hospitalizations among children (30). In a large prospective study of US pneumonias, rhinoviruses have been identified as the second most prevalent etiology of pneumonia in children after respiratory syncytial virus and the first most common etiology among adults (31). There are more than 150 unique types of rhinoviruses. Among the three genotypes (A, B, and C) types A and C are most often associated with increased morbidity and bacterial secondary infection. In animals, rhinovirus type C has been associated with morbidity in chimpanzees (32). With an array of unique serotypes no vaccines or approved antiviral therapies have been commercially produced; however, experiments have suggested that vaccines and antiviral therapy may be possible (33, 34).

### EV D68

Enterovirus D68 has caused sporadic respiratory disease outbreaks across Asia, Europe, and USA since 1960s; however, in 2014, a nationwide outbreak of D68 was associated with severe respiratory illness in USA, resulting in 14 deaths out of a known 1,150 cases (35). The CDC found 36% of all EVs tested during this outbreak were D68 and that patients with a history of asthma were found to be at a disproportionately increased risk of infection (36). One study of the 2014 outbreak found 59% of patients seen with EV-D68 in hospitals across Missouri, Illinois, and Colorado were admitted to intensive care units and 28% received ventilator support (35). In a study evaluating EVs in non-human primates, EV-D68 was detected as a recombinant zoonotic strain (37).

### Enterovirus 71 (EV71)

While there are several strains of coxsackievirus and EVs that can cause hand-foot-and-mouth disease (HFMD), EV71 is most commonly associated with severe disease outcomes. HFMD predominantly affects young children and is found worldwide but especially in the Asia-Pacific region. Although EV71 is not typically detected in animals, recent research has indicated that it infects non-human primates (38). Various antiviral therapies are currently under study, including small molecules, monoclonals, and antivirals. Vaccine candidates are also in development, with two vaccines currently available in China, which involve recombinant proteins, attenuated strains, inactivated whole-virus and virus-like particles, and DNA vaccines (39).

## Human Ad

### Introduction and Epidemiology

First discovered in 1953 by Rowe et al., Ads are non-enveloped, double-stranded DNA viruses with 57 unique serotypes, some of which are specific for attacking the respiratory track, conjunctiva, or gastrointestinal track (40). Key features of Ad infections include various symptoms of disease, including rhinorrhea, nasal congestion, cough, sneezing, pharyngitis, keratoconjunctivitis, pneumonia, meningitis, gastroenteritis, cystitis, and encephalitis. Illnesses may be asymptomatic, mild, or severe; however, immunocompromised patients and infants are at increased risk of severe morbidity and death.

### Ad Outbreaks

Outbreaks of respiratory Ad infection are common in both military recruits and other large training groups, such as police trainees. Large persistent epidemics of Ad type 4-associated respiratory disease have been documented in various military trainees (41–43).

In response to the increased disease burden from Ad4 and Ad7 in military recruits, Teva has made a vaccine available to military recruits in USA (42). Despite a 12-year hiatus from use, in late 2011 oral Ad4 and Ad7 vaccines were reintroduced as an infection control measure for military recruits (42). After reintroduction, military recruits experienced a 100-fold decline in Ad disease burden, which accounted for the prevention of approximately 1 death, 1,100–2,700 hospitalizations, and 13,000 febrile Ad cases per year among trainees (44).

### Emerging Ads

Outbreaks of Ad in the general population have been characterized by infection due to novel viruses such as Ad7h, Ad7d2, Ad14a, and Ad3 variants. These novel viruses are sometimes associated with high attack rates and a high prevalence of pneumonia. Severe mortality is also prevalent among patients with chronic disease and in the elderly.

One of the most important novel serotypes, Ad14, previously rarely reported, is now considered as an emerging Ad type causing severe and sometimes fatal respiratory illness in patients of all ages (45). Beginning in 2005, Ad14 cases were suddenly identified in four locations across USA (46); the strain associated with this outbreak was different than the original Ad14 strain isolated in 1950s. The novel strain, Ad14a, has now spread to numerous US states and is associated with a higher rate of severe illness when compared to other Ad strains.

Novel Ad species have also been recently detected in cross-species infections from non-human primates to man in USA and between psittacine birds and man in China (47). These cross-species infections indicate that Ads should be monitored for their potential to cause cross-species outbreaks. In a recent review of the risks of potential outbreaks associated with zoonotic Ad (48), it was noted that intense human–animal interaction is likely to increase the probability of emergent cross-species Ad infection. Additionally, the recombination of AdVs with latent “host-specific” AdVs is the most likely scenario for adaptation to a new host, either human or animal.

Currently, there are no FDA approved antivirals for Ad infection; however, the best antiviral success has been seen with ribavirin, cidofovir, and most recently brincidofovir an analog of cidofovir (49).

## Conclusion

As it is clear that many emerging respiratory viruses have zoono-tic reservoirs, the design and implementation of effective control strategies are increasingly important. It has been suggested that avoiding direct contact with animals known to be zoonotic reservoirs for these viruses is one potential strategy (50); however, in populations where contact at the human–animal interface is common this may not be an acceptable solution.

Complex disease problems cannot be solved by one institution or one discipline; as such, this presents opportunities to incorporate the One Health approach of working across disciplines to incorporate human, animal, and environmental health to solve complex problems. Although some of the respiratory viruses described here are found almost exclusively in humans (Ad strains), many of the most important emerging respiratory viruses are found at the human/animal interface. This suggests that strategies for novel virus detection should incorporate global surveillance at the human–animal interface to detect potentially emerging zoonotic viruses. This surveillance will require collaboration and cooperation among many stakeholders in order to address emerging and novel viral diseases.

## Author Contributions

EB, JF, and JC conducted the literature review and wrote the manuscript; GG conceived the idea of the review and helped revise the manuscript to add important scientific content and refine the interpretation of the results. All the authors reviewed the final version of the manuscript and agreed to its submission.

## Conflict of Interest Statement

The authors declare that this research was conducted in the absence of any potential financial or commercial conflicts of interest.
